# *O*,*O*′-2-Iodo-1,3-phenyl­ene bis­(diphenyl­phosphinothio­ate)

**DOI:** 10.1107/S1600536811033629

**Published:** 2011-08-27

**Authors:** Jun-long Niu, Xia Wang, Biao Gao, Mao-ping Song

**Affiliations:** a Department of Chemistry, Henan Key Laboratory of Chemical Biology and Organic Chemistry, Zhengzhou University, Zhengzhou, 450052, People’s Republic of China; b Pharmacy College, Henan University of Traditional Chinese Medicine, Zhengzhou, 450008, People’s Republic of China

## Abstract

The title compound, C_30_H_23_IO_2_P_2_S_2_, was synthesized by the reaction of 2-iodo­benzene-1,3-diol, chloro­diphenyl­phosphine, Et_3_N and sulfur. The P=S bonds project to opposite sides of the central aromatic ring. The O—P—S and C—P—S bond angles are significantly larger than the O—P—C and C—P—C bond angles, indicating significant distortion of the tetra­hedral geometries of the P atoms. The P=S bond lengths of 1.9311 (13) and 1.9302 (12) Å in the title compound are shorter than that found in Ph_3_P=S [1.950 (3) Å] because the replacement of one C atom attached the P atom by an O atom increases the effective electronegativity of the P atom.

## Related literature

For related compounds, see: Eisler & Puddephatt (2006 ▶); Aleksanyan *et al.* (2011 ▶); Mague *et al.* (2007 ▶).
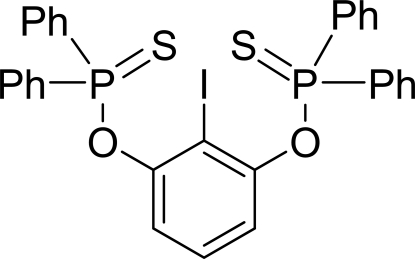


## Experimental

### 

#### Crystal data

C_30_H_23_IO_2_P_2_S_2_
*M* *_r_* = 668.44
Monoclinic, 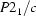

*a* = 12.5467 (11) Å
*b* = 13.4389 (9) Å
*c* = 18.0010 (13) Å
β = 108.299 (8)°
*V* = 2881.7 (4) Å^3^
*Z* = 4
Mo *K*α radiation
μ = 1.39 mm^−1^
*T* = 293 K
0.2 × 0.2 × 0.15 mm

#### Data collection

Oxford Diffraction Xcalibur Eos Gemini diffractometer
Absorption correction: multi-scan (*CrysAlis PRO*; Agilent, 2011 ▶) *T*_min_ = 0.739, *T*_max_ = 1.000
13529 measured reflections
5949 independent reflections
4846 reflections with *I* > 2σ(*I*)
*R*_int_ = 0.029

#### Refinement

*R*[*F* ^2^ > 2σ(*F* ^2^)] = 0.036
*wR*(*F* ^2^) = 0.086
*S* = 1.08
5949 reflections
335 parameters
H-atom parameters constrained
Δρ_max_ = 0.46 e Å^−3^
Δρ_min_ = −0.66 e Å^−3^

### 

Data collection: *CrysAlis PRO* (Agilent, 2011 ▶); cell refinement: *CrysAlis PRO*; data reduction: *CrysAlis PRO*; program(s) used to solve structure: *SHELXS97* (Sheldrick, 2008 ▶); program(s) used to refine structure: *SHELXL97* (Sheldrick, 2008 ▶); molecular graphics: *OLEX2* (Dolomanov *et al.*, 2009 ▶); software used to prepare material for publication: *OLEX2*.

## Supplementary Material

Crystal structure: contains datablock(s) global, I. DOI: 10.1107/S1600536811033629/hb6374sup1.cif

Structure factors: contains datablock(s) I. DOI: 10.1107/S1600536811033629/hb6374Isup2.hkl

Additional supplementary materials:  crystallographic information; 3D view; checkCIF report

## Figures and Tables

**Fig. 1.**
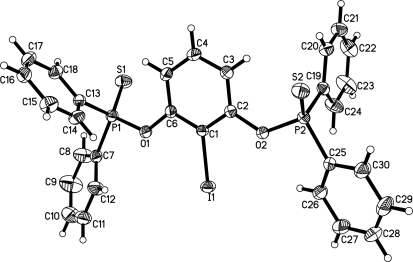
View of the title compound, showing 30% probability ellipsolids.

**Fig. 2.**
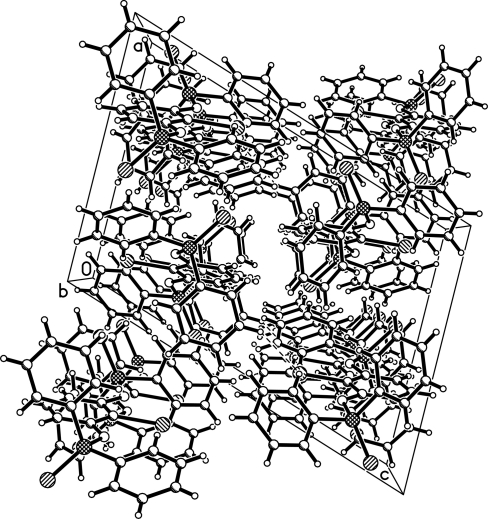
A view of the crystal packing along the b axis.

## supplementary crystallographic information

### Comment

Phosphinothioates play significant roles in coordination chemistry and transition-metal catalysis (Eisler & Puddephatt, 2006). Furthermore, the ability of thiophosphinoyl moieties to act as bridging ligands has prompted the development of the pincer-type chemistry (Aleksanyan *et al.*, 2011). In this work, through a facile one-pot phosphorylation/oxidiation procedure, we obtained the title compound, which is reported here. The title compound, C_30_H_23_IO_2_P_2_S_2_, was synthesized by the reaction of 2-iodobenzene-1,3-diol, chlorodiphenylphosphine, Et_3_N with sulfur. The compound exhibits distorted tetrahedral geometry about the P1 and P2 atoms (Fig. 1), and the O—P—S, C—P—S bond angles are significantly larger than the O—P—C, C—P—C bond angles. The P=S bonds of 1.9311 (13) and 1.9302 (12) Å are shorter than that found in Ph_3_P=S [1.950 (3) Å] because the replacement of one carbon on phosphorus by oxygen increases the effective electronegativity of the P atom.

### Experimental

A mixture of 2-iodobenzene-1,3-diol (118 mg, 0.5 mmol), Et_3_N (0.2 ml, 1.5 mmol) and chlorodiphenylphosphine (0.14 ml, 0.75 mmol) in toluene (5 ml) was heated to reflux for 3 h. Then sulfur (48 mg, 1.5 mmol) was added and the mixture was heated to 90\ %C for 30 min. The product was isolated and recrystallized from dicholomethane/hexane, colorless crystals of the title compound was obtained.

### Crystal data

**Table:**

C_30_H_23_IO_2_P_2_S_2_	*F*(000) = 1336
*M**_r_* = 668.44	*D*_x_ = 1.541 Mg m^−^^3^
Monoclinic, *P*2_1_/*c*	Mo *K*α radiation, λ = 0.7107 Å
*a* = 12.5467 (11) Å	Cell parameters from 4397 reflections
*b* = 13.4389 (9) Å	θ = 3.0–29.1°
*c* = 18.0010 (13) Å	µ = 1.39 mm^−^^1^
β = 108.299 (8)°	*T* = 293 K
*V* = 2881.7 (4) Å^3^	Prismatic, colorless
*Z* = 4	0.2 × 0.2 × 0.15 mm

### Data collection

**Table:**

Agilent Xcalibur Eos Gemini diffractometer	5949 independent reflections
Radiation source: Enhance (Mo) X-ray Source	4846 reflections with *I* > 2σ(*I*)
graphite	*R*_int_ = 0.029
Detector resolution: 16.2312 pixels mm^-1^	θ_max_ = 26.5°, θ_min_ = 3.0°
ω scans	*h* = −14→15
Absorption correction: multi-scan (*CrysAlis PRO*; Agilent Technologies, 2011)	*k* = −16→12
*T*_min_ = 0.739, *T*_max_ = 1.000	*l* = −20→22
13529 measured reflections	

### Refinement

**Table:**

Refinement on *F*^2^	Secondary atom site location: difference Fourier map
Least-squares matrix: full	Hydrogen site location: inferred from neighbouring sites
*R*[*F*^2^ > 2σ(*F*^2^)] = 0.036	H-atom parameters constrained
*wR*(*F*^2^) = 0.086	*w* = 1/[σ^2^(*F*_o_^2^) + (0.0312*P*)^2^ + 1.3829*P*] where *P* = (*F*_o_^2^ + 2*F*_c_^2^)/3
*S* = 1.08	(Δ/σ)_max_ = 0.001
5949 reflections	Δρ_max_ = 0.46 e Å^−^^3^
335 parameters	Δρ_min_ = −0.66 e Å^−^^3^
0 restraints	Extinction correction: *SHELXL*, Fc^*^=kFc[1+0.001xFc^2^λ^3^/sin(2θ)]^-1/4^
Primary atom site location: structure-invariant direct methods	Extinction coefficient: 0.00127 (16)

### Special details

**Table:**

Geometry. All e.s.d.'s (except the e.s.d. in the dihedral angle between two l.s. planes) are estimated using the full covariance matrix. The cell e.s.d.'s are taken into account individually in the estimation of e.s.d.'s in distances, angles and torsion angles; correlations between e.s.d.'s in cell parameters are only used when they are defined by crystal symmetry. An approximate (isotropic) treatment of cell e.s.d.'s is used for estimating e.s.d.'s involving l.s. planes.
Refinement. Refinement of *F*^2^ against ALL reflections. The weighted *R*-factor *wR* and goodness of fit *S* are based on *F*^2^, conventional *R*-factors *R* are based on *F*, with *F* set to zero for negative *F*^2^. The threshold expression of *F*^2^ > σ(*F*^2^) is used only for calculating *R*-factors(gt) *etc*. and is not relevant to the choice of reflections for refinement. *R*-factors based on *F*^2^ are statistically about twice as large as those based on *F*, and *R*- factors based on ALL data will be even larger.

### Fractional atomic coordinates and isotropic or equivalent isotropic displacement parameters (Å^2^)

**Table:**

	*x*	*y*	*z*	*U*_iso_*/*U*_eq_	
I1	0.19393 (2)	0.150065 (17)	0.148128 (12)	0.04653 (10)	
S1	0.07696 (8)	−0.12781 (8)	0.35968 (6)	0.0583 (3)	
S2	0.52162 (7)	0.42008 (7)	0.36702 (5)	0.0485 (2)	
P1	0.16934 (7)	−0.13367 (6)	0.29185 (5)	0.0375 (2)	
P2	0.36544 (7)	0.41688 (6)	0.30439 (5)	0.03448 (19)	
O1	0.20214 (19)	−0.02722 (15)	0.26108 (12)	0.0426 (5)	
O2	0.31642 (19)	0.30818 (15)	0.27040 (12)	0.0420 (5)	
C1	0.2609 (2)	0.1405 (2)	0.26925 (17)	0.0327 (7)	
C2	0.3132 (3)	0.2224 (2)	0.31251 (18)	0.0351 (7)	
C3	0.3597 (3)	0.2160 (2)	0.3933 (2)	0.0461 (8)	
H3	0.3942	0.2711	0.4222	0.055*	
C4	0.3540 (3)	0.1268 (3)	0.4300 (2)	0.0519 (9)	
H4	0.3855	0.1222	0.4840	0.062*	
C5	0.3026 (3)	0.0446 (2)	0.3880 (2)	0.0474 (9)	
H5	0.2997	−0.0150	0.4136	0.057*	
C6	0.2555 (3)	0.0513 (2)	0.30779 (19)	0.0368 (7)	
C7	0.1040 (3)	−0.1909 (2)	0.1982 (2)	0.0399 (7)	
C8	0.0146 (4)	−0.2534 (3)	0.1896 (3)	0.0756 (14)	
H8	−0.0130	−0.2636	0.2313	0.091*	
C9	−0.0347 (4)	−0.3012 (4)	0.1193 (3)	0.0989 (18)	
H9	−0.0945	−0.3445	0.1141	0.119*	
C10	0.0039 (4)	−0.2852 (4)	0.0571 (3)	0.0801 (14)	
H10	−0.0303	−0.3167	0.0095	0.096*	
C11	0.0925 (4)	−0.2230 (3)	0.0651 (2)	0.0680 (12)	
H11	0.1188	−0.2122	0.0229	0.082*	
C12	0.1433 (4)	−0.1762 (3)	0.1353 (2)	0.0579 (10)	
H12	0.2043	−0.1345	0.1404	0.069*	
C13	0.3030 (3)	−0.1930 (2)	0.33497 (18)	0.0356 (7)	
C14	0.3933 (3)	−0.1716 (3)	0.3081 (2)	0.0489 (9)	
H14	0.3847	−0.1264	0.2675	0.059*	
C15	0.4951 (3)	−0.2175 (3)	0.3418 (2)	0.0565 (10)	
H15	0.5553	−0.2027	0.3239	0.068*	
C16	0.5088 (3)	−0.2845 (3)	0.4011 (2)	0.0508 (9)	
H16	0.5777	−0.3157	0.4231	0.061*	
C17	0.4206 (3)	−0.3054 (3)	0.4278 (2)	0.0529 (9)	
H17	0.4301	−0.3507	0.4685	0.063*	
C18	0.3181 (3)	−0.2603 (2)	0.3955 (2)	0.0449 (8)	
H18	0.2589	−0.2751	0.4143	0.054*	
C19	0.2699 (3)	0.4596 (2)	0.35398 (18)	0.0356 (7)	
C20	0.3034 (3)	0.4685 (2)	0.4346 (2)	0.0447 (8)	
H20	0.3772	0.4545	0.4639	0.054*	
C21	0.2278 (3)	0.4980 (3)	0.4715 (2)	0.0572 (10)	
H21	0.2506	0.5028	0.5258	0.069*	
C22	0.1204 (3)	0.5202 (3)	0.4293 (3)	0.0651 (12)	
H22	0.0697	0.5404	0.4546	0.078*	
C23	0.0868 (3)	0.5128 (4)	0.3497 (3)	0.0743 (14)	
H23	0.0130	0.5278	0.3210	0.089*	
C24	0.1605 (3)	0.4835 (3)	0.3112 (2)	0.0591 (11)	
H24	0.1369	0.4797	0.2569	0.071*	
C25	0.3351 (3)	0.4819 (2)	0.21277 (19)	0.0385 (7)	
C26	0.2499 (3)	0.4536 (3)	0.1471 (2)	0.0529 (9)	
H26	0.2061	0.3983	0.1487	0.064*	
C27	0.2295 (4)	0.5079 (3)	0.0784 (2)	0.0693 (12)	
H27	0.1722	0.4884	0.0340	0.083*	
C28	0.2926 (4)	0.5895 (3)	0.0756 (3)	0.0697 (12)	
H28	0.2785	0.6252	0.0293	0.084*	
C29	0.3764 (4)	0.6185 (3)	0.1407 (3)	0.0748 (13)	
H29	0.4186	0.6747	0.1389	0.090*	
C30	0.3990 (3)	0.5647 (3)	0.2097 (2)	0.0595 (10)	
H30	0.4570	0.5842	0.2538	0.071*	

### Atomic displacement parameters (Å^2^)

**Table:**

	*U*^11^	*U*^22^	*U*^33^	*U*^12^	*U*^13^	*U*^23^
I1	0.05978 (17)	0.04644 (15)	0.03023 (13)	−0.01443 (11)	0.00964 (10)	−0.00163 (10)
S1	0.0519 (6)	0.0797 (7)	0.0486 (6)	0.0026 (5)	0.0233 (4)	0.0004 (5)
S2	0.0397 (5)	0.0591 (6)	0.0440 (5)	−0.0006 (4)	0.0093 (4)	−0.0029 (4)
P1	0.0433 (5)	0.0336 (4)	0.0358 (5)	−0.0042 (4)	0.0125 (4)	0.0015 (4)
P2	0.0402 (5)	0.0314 (4)	0.0306 (4)	−0.0035 (3)	0.0094 (3)	−0.0027 (3)
O1	0.0583 (14)	0.0299 (11)	0.0344 (12)	−0.0070 (10)	0.0072 (10)	−0.0007 (10)
O2	0.0622 (15)	0.0297 (11)	0.0307 (12)	−0.0063 (11)	0.0100 (10)	−0.0008 (10)
C1	0.0345 (16)	0.0352 (16)	0.0273 (15)	0.0034 (13)	0.0080 (12)	−0.0005 (13)
C2	0.0403 (17)	0.0308 (16)	0.0341 (17)	−0.0019 (14)	0.0114 (13)	−0.0015 (14)
C3	0.060 (2)	0.0348 (18)	0.0366 (19)	−0.0048 (16)	0.0051 (16)	−0.0025 (15)
C4	0.071 (3)	0.0418 (19)	0.0324 (18)	−0.0017 (18)	0.0019 (17)	0.0019 (15)
C5	0.066 (2)	0.0321 (17)	0.0385 (19)	−0.0002 (17)	0.0078 (16)	0.0056 (15)
C6	0.0410 (18)	0.0301 (16)	0.0363 (17)	0.0015 (14)	0.0077 (14)	−0.0011 (14)
C7	0.0435 (19)	0.0334 (16)	0.0415 (19)	−0.0041 (15)	0.0115 (15)	−0.0001 (15)
C8	0.081 (3)	0.092 (3)	0.059 (3)	−0.046 (3)	0.029 (2)	−0.017 (3)
C9	0.095 (4)	0.119 (4)	0.083 (4)	−0.064 (3)	0.028 (3)	−0.035 (3)
C10	0.087 (3)	0.080 (3)	0.059 (3)	−0.020 (3)	0.002 (2)	−0.025 (3)
C11	0.091 (3)	0.065 (3)	0.048 (2)	−0.010 (2)	0.023 (2)	−0.007 (2)
C12	0.072 (3)	0.056 (2)	0.050 (2)	−0.021 (2)	0.026 (2)	−0.0100 (19)
C13	0.0431 (18)	0.0311 (16)	0.0325 (17)	−0.0061 (14)	0.0117 (14)	−0.0010 (13)
C14	0.051 (2)	0.056 (2)	0.043 (2)	0.0003 (18)	0.0194 (17)	0.0130 (17)
C15	0.043 (2)	0.074 (3)	0.058 (2)	0.0026 (19)	0.0231 (18)	0.006 (2)
C16	0.050 (2)	0.048 (2)	0.048 (2)	0.0067 (17)	0.0075 (17)	0.0000 (18)
C17	0.060 (2)	0.044 (2)	0.046 (2)	−0.0019 (18)	0.0036 (18)	0.0102 (17)
C18	0.048 (2)	0.0415 (18)	0.045 (2)	−0.0076 (16)	0.0138 (16)	0.0072 (16)
C19	0.0350 (17)	0.0344 (16)	0.0371 (18)	−0.0057 (14)	0.0107 (13)	−0.0038 (14)
C20	0.050 (2)	0.0466 (19)	0.0387 (19)	−0.0003 (17)	0.0150 (16)	0.0035 (16)
C21	0.072 (3)	0.062 (2)	0.045 (2)	−0.004 (2)	0.030 (2)	−0.0026 (19)
C22	0.054 (2)	0.073 (3)	0.082 (3)	−0.009 (2)	0.041 (2)	−0.022 (2)
C23	0.036 (2)	0.102 (4)	0.080 (3)	−0.003 (2)	0.013 (2)	−0.037 (3)
C24	0.042 (2)	0.080 (3)	0.048 (2)	0.000 (2)	0.0040 (17)	−0.021 (2)
C25	0.051 (2)	0.0304 (16)	0.0357 (18)	−0.0016 (15)	0.0161 (15)	0.0004 (14)
C26	0.069 (2)	0.044 (2)	0.040 (2)	−0.0105 (19)	0.0086 (17)	0.0030 (17)
C27	0.091 (3)	0.066 (3)	0.039 (2)	−0.004 (2)	0.003 (2)	0.005 (2)
C28	0.104 (4)	0.060 (3)	0.049 (2)	0.009 (3)	0.030 (2)	0.020 (2)
C29	0.098 (4)	0.056 (2)	0.074 (3)	−0.018 (2)	0.031 (3)	0.017 (2)
C30	0.074 (3)	0.052 (2)	0.049 (2)	−0.019 (2)	0.0130 (19)	0.0046 (19)

### Geometric parameters (Å, °)

**Table:**

I1—C1	2.080 (3)	C13—C18	1.383 (4)
S1—P1	1.9311 (13)	C14—H14	0.9300
S2—P2	1.9302 (12)	C14—C15	1.376 (5)
P1—O1	1.633 (2)	C15—H15	0.9300
P1—C7	1.799 (3)	C15—C16	1.366 (5)
P1—C13	1.798 (3)	C16—H16	0.9300
P2—O2	1.628 (2)	C16—C17	1.368 (5)
P2—C19	1.799 (3)	C17—H17	0.9300
P2—C25	1.798 (3)	C17—C18	1.375 (5)
O1—C6	1.384 (4)	C18—H18	0.9300
O2—C2	1.387 (4)	C19—C20	1.383 (4)
C1—C2	1.389 (4)	C19—C24	1.384 (4)
C1—C6	1.397 (4)	C20—H20	0.9300
C2—C3	1.388 (4)	C20—C21	1.377 (5)
C3—H3	0.9300	C21—H21	0.9300
C3—C4	1.381 (5)	C21—C22	1.357 (5)
C4—H4	0.9300	C22—H22	0.9300
C4—C5	1.380 (5)	C22—C23	1.365 (6)
C5—H5	0.9300	C23—H23	0.9300
C5—C6	1.381 (5)	C23—C24	1.375 (5)
C7—C8	1.371 (5)	C24—H24	0.9300
C7—C12	1.385 (5)	C25—C26	1.375 (5)
C8—H8	0.9300	C25—C30	1.382 (5)
C8—C9	1.381 (6)	C26—H26	0.9300
C9—H9	0.9300	C26—C27	1.388 (5)
C9—C10	1.370 (6)	C27—H27	0.9300
C10—H10	0.9300	C27—C28	1.363 (6)
C10—C11	1.361 (6)	C28—H28	0.9300
C11—H11	0.9300	C28—C29	1.363 (6)
C11—C12	1.375 (5)	C29—H29	0.9300
C12—H12	0.9300	C29—C30	1.388 (5)
C13—C14	1.395 (4)	C30—H30	0.9300
			
O1—P1—S1	116.36 (10)	C18—C13—C14	118.9 (3)
O1—P1—C7	98.39 (13)	C13—C14—H14	120.0
O1—P1—C13	103.58 (13)	C15—C14—C13	119.9 (3)
C7—P1—S1	114.98 (12)	C15—C14—H14	120.0
C13—P1—S1	114.12 (11)	C14—C15—H15	119.6
C13—P1—C7	107.69 (15)	C16—C15—C14	120.7 (3)
O2—P2—S2	115.72 (10)	C16—C15—H15	119.6
O2—P2—C19	103.94 (13)	C15—C16—H16	120.2
O2—P2—C25	98.35 (13)	C15—C16—C17	119.6 (3)
C19—P2—S2	114.56 (11)	C17—C16—H16	120.2
C19—P2—C25	108.52 (15)	C16—C17—H17	119.6
C25—P2—S2	114.13 (11)	C16—C17—C18	120.9 (3)
C6—O1—P1	126.0 (2)	C18—C17—H17	119.6
C2—O2—P2	127.78 (19)	C13—C18—H18	120.0
C2—C1—I1	120.2 (2)	C17—C18—C13	120.0 (3)
C2—C1—C6	119.3 (3)	C17—C18—H18	120.0
C6—C1—I1	120.5 (2)	C20—C19—P2	121.2 (2)
O2—C2—C1	116.2 (3)	C20—C19—C24	119.0 (3)
O2—C2—C3	123.3 (3)	C24—C19—P2	119.8 (3)
C1—C2—C3	120.5 (3)	C19—C20—H20	119.9
C2—C3—H3	120.4	C21—C20—C19	120.2 (3)
C4—C3—C2	119.2 (3)	C21—C20—H20	119.9
C4—C3—H3	120.4	C20—C21—H21	119.8
C3—C4—H4	119.4	C22—C21—C20	120.4 (4)
C5—C4—C3	121.2 (3)	C22—C21—H21	119.8
C5—C4—H4	119.4	C21—C22—H22	120.1
C4—C5—H5	120.2	C21—C22—C23	119.8 (4)
C4—C5—C6	119.6 (3)	C23—C22—H22	120.1
C6—C5—H5	120.2	C22—C23—H23	119.5
O1—C6—C1	116.2 (3)	C22—C23—C24	121.0 (4)
C5—C6—O1	123.5 (3)	C24—C23—H23	119.5
C5—C6—C1	120.3 (3)	C19—C24—H24	120.3
C8—C7—P1	118.9 (3)	C23—C24—C19	119.5 (4)
C8—C7—C12	118.9 (4)	C23—C24—H24	120.3
C12—C7—P1	122.1 (3)	C26—C25—P2	122.5 (3)
C7—C8—H8	119.9	C26—C25—C30	119.5 (3)
C7—C8—C9	120.3 (4)	C30—C25—P2	118.0 (3)
C9—C8—H8	119.9	C25—C26—H26	120.1
C8—C9—H9	119.8	C25—C26—C27	119.8 (3)
C10—C9—C8	120.3 (4)	C27—C26—H26	120.1
C10—C9—H9	119.8	C26—C27—H27	119.7
C9—C10—H10	120.1	C28—C27—C26	120.6 (4)
C11—C10—C9	119.8 (4)	C28—C27—H27	119.7
C11—C10—H10	120.1	C27—C28—H28	120.1
C10—C11—H11	119.8	C29—C28—C27	119.8 (4)
C10—C11—C12	120.4 (4)	C29—C28—H28	120.1
C12—C11—H11	119.8	C28—C29—H29	119.8
C7—C12—H12	119.8	C28—C29—C30	120.5 (4)
C11—C12—C7	120.3 (4)	C30—C29—H29	119.8
C11—C12—H12	119.8	C25—C30—C29	119.8 (4)
C14—C13—P1	120.6 (2)	C25—C30—H30	120.1
C18—C13—P1	120.6 (2)	C29—C30—H30	120.1
			
I1—C1—C2—O2	0.5 (4)	C4—C5—C6—O1	−179.9 (3)
I1—C1—C2—C3	−179.0 (2)	C4—C5—C6—C1	0.7 (5)
I1—C1—C6—O1	−1.1 (4)	C6—C1—C2—O2	179.4 (3)
I1—C1—C6—C5	178.3 (3)	C6—C1—C2—C3	0.0 (5)
S1—P1—O1—C6	56.1 (3)	C7—P1—O1—C6	179.5 (3)
S1—P1—C7—C8	−21.0 (4)	C7—P1—C13—C14	75.2 (3)
S1—P1—C7—C12	161.0 (3)	C7—P1—C13—C18	−105.1 (3)
S1—P1—C13—C14	−155.9 (2)	C7—C8—C9—C10	−1.2 (9)
S1—P1—C13—C18	23.9 (3)	C8—C7—C12—C11	0.6 (6)
S2—P2—O2—C2	58.9 (3)	C8—C9—C10—C11	1.0 (9)
S2—P2—C19—C20	−15.9 (3)	C9—C10—C11—C12	0.0 (8)
S2—P2—C19—C24	164.6 (3)	C10—C11—C12—C7	−0.8 (7)
S2—P2—C25—C26	149.2 (3)	C12—C7—C8—C9	0.4 (7)
S2—P2—C25—C30	−32.3 (3)	C13—P1—O1—C6	−70.0 (3)
P1—O1—C6—C1	−171.1 (2)	C13—P1—C7—C8	107.4 (3)
P1—O1—C6—C5	9.5 (5)	C13—P1—C7—C12	−70.6 (3)
P1—C7—C8—C9	−177.6 (4)	C13—C14—C15—C16	0.4 (6)
P1—C7—C12—C11	178.6 (3)	C14—C13—C18—C17	−0.4 (5)
P1—C13—C14—C15	179.9 (3)	C14—C15—C16—C17	−0.7 (6)
P1—C13—C18—C17	179.8 (3)	C15—C16—C17—C18	0.4 (6)
P2—O2—C2—C1	176.5 (2)	C16—C17—C18—C13	0.1 (5)
P2—O2—C2—C3	−4.1 (5)	C18—C13—C14—C15	0.1 (5)
P2—C19—C20—C21	−177.7 (3)	C19—P2—O2—C2	−67.6 (3)
P2—C19—C24—C23	177.8 (3)	C19—P2—C25—C26	−81.7 (3)
P2—C25—C26—C27	178.9 (3)	C19—P2—C25—C30	96.8 (3)
P2—C25—C30—C29	−178.3 (3)	C19—C20—C21—C22	−1.1 (6)
O1—P1—C7—C8	−145.4 (3)	C20—C19—C24—C23	−1.8 (6)
O1—P1—C7—C12	36.6 (3)	C20—C21—C22—C23	0.3 (6)
O1—P1—C13—C14	−28.4 (3)	C21—C22—C23—C24	−0.3 (7)
O1—P1—C13—C18	151.4 (3)	C22—C23—C24—C19	1.0 (7)
O2—P2—C19—C20	111.3 (3)	C24—C19—C20—C21	1.8 (5)
O2—P2—C19—C24	−68.2 (3)	C25—P2—O2—C2	−179.2 (3)
O2—P2—C25—C26	26.1 (3)	C25—P2—C19—C20	−144.7 (3)
O2—P2—C25—C30	−155.4 (3)	C25—P2—C19—C24	35.7 (3)
O2—C2—C3—C4	−178.9 (3)	C25—C26—C27—C28	−0.4 (7)
C1—C2—C3—C4	0.5 (5)	C26—C25—C30—C29	0.3 (6)
C2—C1—C6—O1	180.0 (3)	C26—C27—C28—C29	−0.3 (7)
C2—C1—C6—C5	−0.6 (5)	C27—C28—C29—C30	0.9 (7)
C2—C3—C4—C5	−0.4 (6)	C28—C29—C30—C25	−0.9 (7)
C3—C4—C5—C6	−0.2 (6)	C30—C25—C26—C27	0.4 (6)
